# So Different, yet So Similar: Meta-Analysis and Policy Modeling of Willingness to Participate in Clinical Trials among Brazilians and Indians

**DOI:** 10.1371/journal.pone.0014368

**Published:** 2010-12-16

**Authors:** Guilherme Zammar, Henrique Meister, Jatin Shah, Amruta Phadtare, Luciana Cofiel, Ricardo Pietrobon

**Affiliations:** 1 Pontifícia Universidade Católica do Paraná (PUCPR), Curitiba, Paraná, Brazil; 2 Research on Research Group, Duke University, Durham, North Carolina, United States of America; 3 Duke National University of Singapore (NUS) Graduate Medical School, Singapore, Singapore; 4 Kalpavriksha Healthcare and Research, Thane, India; 5 Department of Surgery, Duke University, Durham, North Carolina, United States of America; Universidad Peruana Cayetano Heredia, Peru

## Abstract

**Background:**

With the global expansion of clinical trials and the expectations of the rise of the emerging economies known as BRICs (Brazil, Russia, India and China), the understanding of factors that affect the willingness to participate in clinical trials of patients from those countries assumes a central role in the future of health research.

**Methods:**

We conducted a systematic review and meta-analysis (SRMA) of willingness to participate in clinical trials among Brazilian patients and then we compared it with Indian patients (with results of another SRMA previously conducted by our group) through a system dynamics model.

**Results:**

Five studies were included in the SRMA of Brazilian patients. Our main findings are 1) the major motivation for Brazilian patients to participate in clinical trials is altruism, 2) monetary reimbursement is the least important factor motivating Brazilian patients, 3) the major barrier for Brazilian patients to not participate in clinical trials is the fear of side effects, and 4) Brazilian patients are more likely willing to participate in clinical trials than Indians.

**Conclusion:**

Our study provides important insights for investigators and sponsors for planning trials in Brazil (and India) in the future. Ignoring these results may lead to unnecessary fund/time spending. More studies are needed to validate our results and for better understanding of this poorly studied theme.

## Introduction

With the current expansion of clinical trials around the globe, the importance of better understanding patients motivations across different cultures and countries is exponentially increased. Assuming that “everyone should think like me” is no longer an assumption that researchers from developed countries can make, the cross-cultural motives being diverse and essential not only for adequate recruitment but also to adjust to local beliefs and moral values. Although there is a substantial amount of literature on factors contributing to participation in trials in America [1]–[4], little is known about this information in developing countries.

The desire to help others is a frequent reason for participation in studies conducted in developed countries. “Altruism” and “opportunity to help others” are cited as reasons in several studies. Personal reasons such as health benefits, are less common [1]–[3]. An important factor that hinders the participation in studies is the memory of traumatic experience, such as Tuskegee Study. The minorities in developed countries are particularly affected by this factor[5]. Often these groups also report the fear of their being used as guinea pigs by the dominant class[1].

The few studies conducted in developing countries show similar reasons, but those related to personal benefits are more common [6]–[10]. A recent meta-analysis involving studies on Indian patients showed that almost half of the patients involved in the study wanted to participate in clinical studies for reasons such as free treatment and improvement of their symptoms.[11]

The objective of this study is therefore to conduct a systematic review and meta analysis of the literature regarding willingness to participate in clinical trials among individuals in Brazil, and then compare these findings through a dynamic model of a similar study previously conducted by our group regarding willingness to participate in clinical trials among individuals in India. [11]

## Results

### Systematic Review

**Figure 1:**
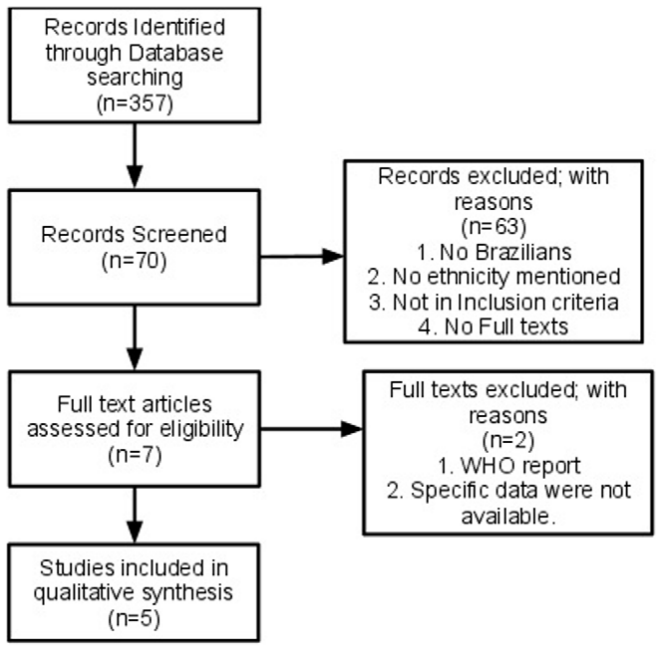
Flowchart with inclusion and exclusion of articles.

The initial review of the literature resulted in 28119 articles, and we selected 357 as relevant and excluded 27762 after reading their titles. After reading the abstracts of the 357 relevant articles, we excluded 287 of them and selected 70 as relevant. After retrieving the full text of these 70 articles, we excluded 63 of them because one of the following reasons: 1) they did not have Brazilian patients, or 2) the ethnicity of the participants were not mentioned, or 3) they did not fit our inclusion criteria, or 4) lack of availability of full text. From the remaining seven studies, we excluded more two: one by being a report from World Health Organization with insufficient data to be analyzed, and other due to absence of needed data at the article and unresponsiveness of the author. This flow chart is summarized in Figure 1. The final list of five studies matching our inclusion and exclusion criteria is described in Table S1. Three of the seven contacted authors replied to our request with no new articles. No discrepancies were noted by the blinded search and the articles found by the blinded reviewer were the same of the ones found by the other reviewers. Observer agreement among the two reviewers (GZ and HM) in relation to the literature search results of title, abstract as well as full-text eligibility were 66.7%, 28.6%, 50% respectively. The literature search results of title and full text show moderate agreement and the abstract show poor agreement.

Out of the five studies included in our analysis, three were conducted in Rio de Janeiro - RJ [8]–[10], one in Belo Horizonte – MG [7] and one in Salvador – BA[6]. Three of the five studies are focused in patient participation in HIV vaccine trials (two in Rio de Janeiro – RJ[9], [10] and one in Belo Horizonte - MG [7]. The study from Salvador - BA were focused in young women participation in human papillomavirus vaccination trials. The remaining study from Rio de Janeiro - RJ [8] were focused in patient participation in general. The age of patients included range from 16 to 50, but one study [8] did not reported the age group. The total number of participants included in our analysis were 2920 (2024 males and 896 females) and more details can be visualized in Table S1.

The results from our systematic review and meta-analysis were subdivided into two groups: Factors favoring participation in clinical trials (Table S2) and factors serving as barrier to participation in clinical trials (Table S3). For the factors favoring participation, we found four main themes (personal health benefits, altruism, convenience and monetary reimbursement) which can be seen with their respective percentages in Table S2. For the factors serving as barrier, we also found four themes (fear of adverse events, inconvenience, mistrust and lack of knowledge) and more details can be seen at table S3.

### Factors favoring participation in clinical trials

#### Altruism 55%

Altruism means “unselfish regard for or devotion to the welfare of others”[12]. In our study, altruism is figuring as the main theme influencing Brazilian patients to participate in clinical trials. Altruism appears as a decisive factor in four out of the five articles analyzed. The reasons cited into the articles related to altruism included the possibility both to benefit others and the opportunity to help science.

#### Personal Health Benefits 30%

The personal benefits to health were a common factors to all articles, is the second most important factor favoring participation in clinical trials. In this case, several reasons were interpreted as benefits to their health. Some patients were interested in the possibility of consultations with specialists, a more detailed consultation with the same doctor or even the ability to consult, because there is no health service in her city. Other interest was related to the possibility of know more about their disease. The possibility of free benefits like HIV-test, snacks or bus ticket were also cited.

#### Convenience 11%

Reasons related to convenience were cited only in two articles. The possibility of not having to wait long for consultation, access to drugs and tests for free were the reasons reported in the articles related to convenience.

#### Monetary Reimbursement 6%

The monetary benefit was quoted only in one article, and is the least important factor favoring the willingness to participate in clinical trials among Brazilian patients. Surprisingly, it was one of the least cited factors, even in a study that involved only patients with low income and analyzing citizens of a developing country.

It should be noted that the sum of the percentage values of factors favoring participation in clinical trial does not equate to 100% as the patients were not limited to report one theme.

### Factors serving as barrier to participation in clinical trials

#### Fear of Adverse Events 12%

The fear of side effects was quoted in three of the five included studies and is the main factor serving as barrier to participation in clinical trials according to Brazilian patients. Besides the fear of the vaccine itself, some patients cited fears that the vaccine could infect them with HIV or induce that serological tests become positive.

#### Inconvenience 2%

Situations considered inconvenient were the less common reasons for not participating in the studies. Reasons like: “*clinic is too far from home*” and “*need to get injections*” were cited in one study and was related to inconvenience.

#### Mistrust 6%

The mistrust factor was present in three studies. In one of them about one third of patients interviewed said they were insecure and needed more information on the subject before deciding. The fear of being used as guinea pigs appeared in 2 other articles. Other reasons included: believe the vaccine will fail, do not trust in government, in drug companies, in the United States or in research scientists.

#### Lack of knowledge 4%

Lack of knowledge was a reason for not participating in clinical trials quoted only in one of the articles. In that study, the refuse was based on the fact of not having sufficient information about the vaccine. It should be noted that, the sum of the percentage values of factors serving as barriers to participation in clinical trial does not equate to 100% as the patients were not limited to report one theme

### Policy model

The baseline model was then simulated with two different types of parameters: one for Brazilian patients and the other for Indian patients. Those parameters were the values for each element that affect ‘Motivations to participate’ and ‘Barriers to participate’, and were obtained from our meta-analysis and from the meta-analysis for Indian patients conducted by our group[11] Those values were summarized in tables S4 and S5.

**Figure 2:**
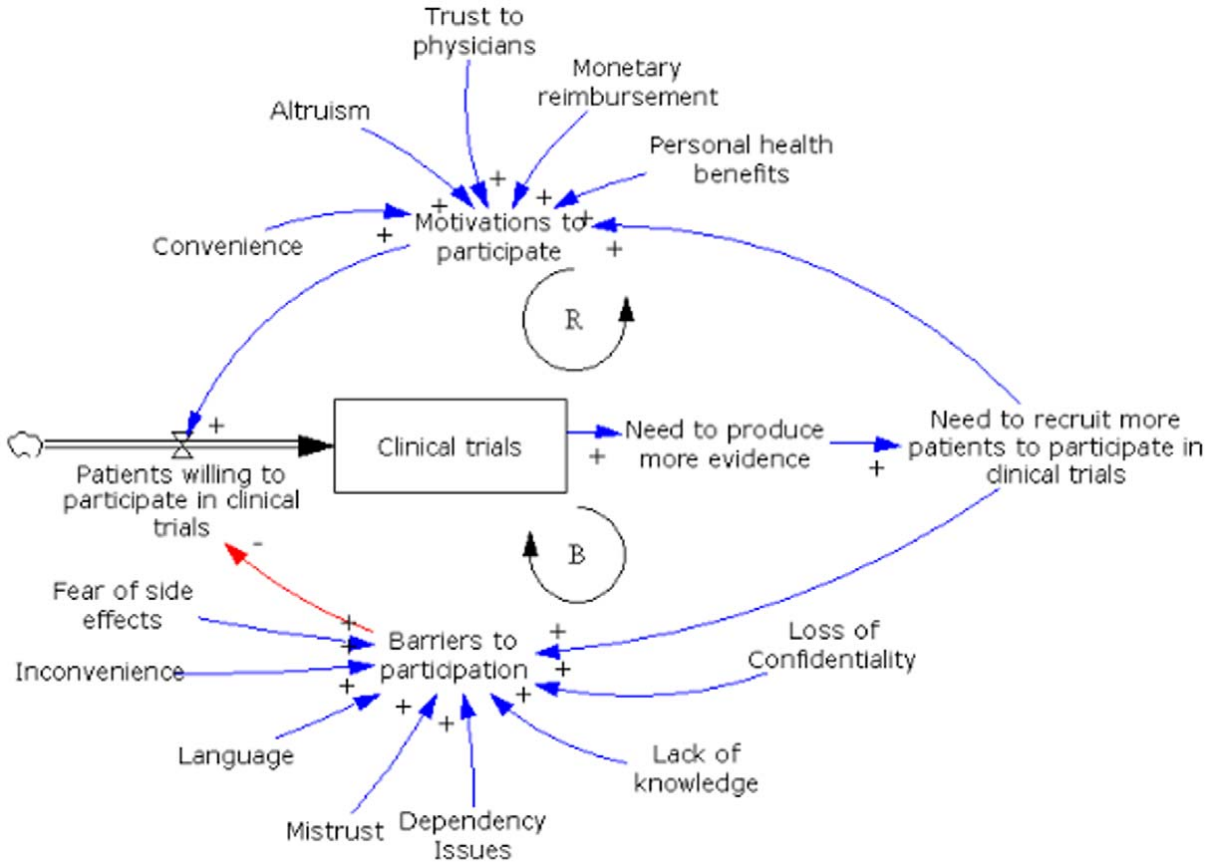
System Dynamics model.

After running the simulation for both scenarios (Brazilian patients versus Indian patients), we graphically displayed the number of clinical trials performed under the two different conditions (Figure 2). To do justice that this model is not explicitly predicting the amount of trials but rather representing the behavior, we don't provide numerical results.

## Discussion

To our knowledge, this is the first study reporting results from a systematic review and meta-analysis of factors that affects the willingness to participate (WTP) in clinical trials for Brazilian patients. We also believe that it is the first study reporting a system dynamics model to compare WTP in clinical trials among Brazilians and Indians. With the expectations of the rise of the emerging economies known as BRIC (Brazil, Russia, India and China)[13], allied with the knowledge of under-representation of some ethnic and minority groups in clinical trials[14]–[16], this study assumes a central role in the future of health research. Our main results are 1) the major motivation for Brazilian patients to participate in clinical trials is altruism, 2) monetary reimbursement is the least important factor motivating Brazilian patients, 3) the major barrier for Brazilian patients not to participate in clinical trials is the fear of side effects, and 4) Brazilian patients are more likely to be willing to participate in clinical trials than Indians.

Altruism is present as a motivational factor for participation in clinical trials in many studies conducted in developed countries [1], [2], [17], [18]. The Brazilian patients involved in this study also cited this as an important motivational factor. The ideas involved in this factor, such as the possibility of helping the community and benefit others in the future, were similar to those found in previous studies[19], [4], [20]. Many of the Brazilian patients were in situations related to HIV and even previous studies related to other diseases have shown the positive influence of altruism.[2], [3], [4] As the desire to “help others” is a common reason to participate in medical research, recruitment strategies for clinical trials highlighting this aspect should yield good results.

The influence of monetary incentives to participate in clinical trials have been reported by previous studies. [21]–[24] A majority of Brazilian patients included in this study have some unique characteristics: homosexual men, intention to participate in HIV-vaccine trials and citizens of a developing country. All these characteristics were previously reported as factors related to the influence of monetary incentives in the enrollment to participate in clinical trials.[25]–[29], but surprisingly in our results that factor is the least important. On the other hand, too much monetary compensation was related to increase concerns with respect to the safety of the research. [30] In patients with other predominant characteristics in contrast to gay men from emerging countries, the influence of monetary incentives vary from positively influencing in some groups [31] and having minimal influence to others.[32] Anyway, the monetary reimbursement strategy seems to be effective to increase the WTP some populations but not in the studied Brazilian patients. Some ethical concerns have to be analyzed when considering to adopt that strategy in order to increase the WTP in clinical trials.[33]

The fear of side effects was broadly reported as a key factor limiting the adherence to medical treatments [34]–[37] and the willingness to accept vaccines. [37], [38] Regarding the willingness to participate in trials, it was also reported as an important serving as a barrier to the willingness [39], [40], and it appears as the main barrier in some studies [41] like we are seeing in our results. For Brazilian patients this fear is the main factor serving as a barrier to participate in clinical trials, and is the second more important for Indians', reinforcing what is seen in literature.

When comparing the WTP of Brazilians' with the Indians' [11] through a system dynamics model, we can see that the Brazilian patients are more willing than Indians' to participate in clinical trials. It can be explained by the lower quantity of factors serving as barrier for Brazilians' compared to Indians'. In the hypothetical scenario within the SD model, we compared the relative quantity of clinical trials that would be generated over time with the same amount of resources. The model then is showing us that the number of clinical trials generated within Brazilian patients would be greater than the generated with Indian patients.

Another interesting point is that 48% of the Indian patients reported the personal health benefits as the major factor influencing the WTP while Brazilian patients reported only 29%. A plausible explanation for that fact may rely on the healthcare system of both countries. In Brazil, there is a publicly-funded universal healthcare system (Sistema Único de Saúde - SUS, portuguese for Unified Health System). [42] In this case, any Brazilian patient already have full healthcare support for free, making personal health benefits not much attractive to them as a factor influencing WTP in clinical trials. The same doesn't occur in India, where the healthcare system is different than what is found in Brazil, making personal health benefits more attractive for Indian patients.

We found moderate and poor agreement among the two reviewers in context to their search results in the same set of databases using the same set of keywords. Since analysis of database search results is a qualitative process the results might differ. Additionally we noted that the results from the blinded reviewer was similar to the other two reviewers thus validating their analysis.

Despite our innovative results, this study has limitations. First, three of the five studies have evaluated the willingness of homosexual men to participate in HIV-vaccine trials. The limitations of it is the risk that these results don't represent the whole Brazilian population. In order to validate the meta-analysis results, our plan is to continue this research project with a multi-center survey including 200 randomly selected outpatients. Second, a system dynamics model is an experimental method, and its results cannot consider random and/or unexpected events. Another limitation related to the SD model is about the fact that the data included in the SD model were not reported in all the 5 trials as reported in table S1. Consequently, the model outputs just represents the behavior of a system created with the data given by us. In this case, we based our inputs totally from the literature based on the two systematic reviews involving the Brazilian and Indian patients. Thirdly, we could not adjust for the differences existing between the articles included in our study due to limitations posed by meta analysis study design. These differences include differences in population, sample size, study objectives, outcomes of interest and data capture.

Our conclusion is that investigators and sponsors must consider our results when planning clinical trials to be performed in Brazil (and India). Based in our results, the best way to incentive Brazilian patients to participate in clinical trials is by making them understand the altruistic side of the trial rather than trying to give monetary incentives. Another essential point is to explain to the patients about possible side effects. Ignore these results may lead to unnecessary fund/time spending. More studies are needed to validate our results and for better understanding of this poorly studied theme.

## Methods

### Systematic Review and Meta-analysis

The objective of the systematic reviewing is to address the research question “which factors influence Brazilian patients to participate in clinical trials?”.

#### Search Strategy

A systematic search was conducted by two reviewers (GRZ, HSM) independently on the following online databases: Pubmed (1985 to 2008), Cochrane (1983 to 2009), CINAHL, the Cumulative Index to Nursing and Allied Health Literature (1985 to 2008), LILACS, Latin American and Caribbean Health Sciences (1982 to 2009) and ‘SciELO Brazil’, Scientific Electronic Library Online (1982 to 2009). LILACS is a biomedical database with articles from Latin America and ‘SciELO Brazil’ is a scientific database with articles from Brazil.

We used a search strategy combining the following keywords (Appendix) relevant to our research question. The search was restricted to studies published in English or Portuguese languages, conducted in adult human subjects. The reviewers (GRZ, HSM) working with the Latin American databases were fluent in Portuguese and Spanish.

Article reference lists and articles listed under the “related articles” link in PubMed were also examined for additional articles. Finally, we subscribed to RSS (real simple syndication) feeds corresponding to each of the search strategies that we had devised and implemented in online databases to track new studies published after we completed the literature review.

#### Selection

We defined selection criteria to filter and shortlist study articles that would qualify for the meta synthesis. Both reviewers (GRZ and HSM) independently evaluated the study articles that were identified based on our search strategy. When there was disagreement about article inclusion, it was resolved by consensus. For inclusion in the SRMA, a study had to meet the following criteria: 1. Involving subjects confined to Brazil (subjects residing in Brazil or of Brazilian origin); 2. Using experimental (trials) or qualitative methods (interviews, focus groups, ethnographic studies, or surveys) to collect data; 3. Studies whose outcome measures included factors affecting participation of Brazilian subjects in clinical trials, and 4. Availability of full text articles. We excluded studies that retrospectively analyzed clinical trial data, studies that evaluated other Latin American populations, unpublished articles, dissertations, and abstracts without full text. We calculated observer agreement for the literature search carried out by the two reviewers (GRZ, HSM).

#### Hand search

We classified the initial list of articles according to the journal in which they were published, so that we could then identify journals that had published most of the articles in our list.

Since three out of five included studies were related to HIV/AIDS, and two of them were published in ‘JAIDS Journal of Acquired Immune Deficiency Syndromes’, we considered JAIDS as a key journal and performed a manual hand search through each issue of that journal for a period ranging from Feb 1988 to Sep 2009.

#### Communication with authors

To confirm that we had identified and retrieved all relevant studies, we communicated through email with the corresponding authors of shortlisted articles to inquire about the existence of any other published studies related to our research question.

#### Validity assessment

To evaluate the reproducibility of our search, an independent blinded search was performed by one of us (AP) who focused only on the inclusion and exclusion criteria.

#### Data abstraction and Study characteristics

All three reviewers (GRZ, HSM and AP) independently collected qualitative and descriptive data from the included studies into a spreadsheet. All data were split into specific headings including: aim, study design, study period, eligibility criteria, geographic location, population characteristics, source of participants, number of participants, data analysis, outcome measures.

#### Percentage retrieval

We extracted data related to the number of participants who contributed to each factor serving as barrier or motivation to participate in clinical trials and the total number of participants in each study. For the studies reporting the number of responses as percentage values, we converted that value into number through simple mathematics (percent value/100 *total number of participants). The total number of participants contributing to each factor were then summed and percentages were calculated for each factor based on total number of respondents. The final results were then summarized in two tables: factors favoring participation (Table S2) and factors serving as barrier to participation (Table S3). Two more tables were created to compare factors from Brazilians with Indians (Tables S4 and S5), based on results obtained from our study for Brazilian patients and from the study for Indian patients [11].

### Modeling

In order to summarize and organize the study findings and compare results from Brazilians with Indians, we used a System Dynamics (SD) [43] approach. SD can be considered as a set of tools that help in understanding a complex system's behavior over time. Since the process of willingness to participate in clinical trials involves multiple components that interact with each other, it can be considered a complex system. [44] In this way, a SD model helps to understand the whole behavior of that system, to predict their behaviour over time, and to compare different groups (such as Brazilians and Indians). For example, these kind of analysis are increasingly used in healthcare research in fields like healthcare policy to plan cardiovascular disease interventions[45], the spread of influenca virus [46] and in Neurosciences to investigate bimanual coordination after strokes.[47]

In our project the SD model had the role of summarizing the main findings in a causal model and corresponding predicted time trends that would result under those assumptions. It was not our intent to provide quantitative predictions. Therefore we have refrained from adding explicit values on the Y-axis in Figure 2 to highlight this goal.

The SD model is graphically represented mainly by stocks (boxes), flows (thick arrows) and variables defining causal loops (thin arrows). Stocks represents variables that accumulate and deplete over time, and they are regulated by a flow. In addition, causal loops were used to create relationships among model elements through feedback loops which were classified as ‘balancing’ (which promotes the balance of the system) or ‘reinforcing’ loops (which promotes growth of the system). The +/− sign at the end of arrows indicate a positive or negative effect, respectively. After analyzing the results of both meta-analyses, a preliminary model was created by one of us (GRZ) using the program Vensim DSS 5.9c for Windows.[48] This is a simulation software made by Ventana Systems, Inc. (Harvard, Massachusetts [49]). This baseline model (Figure 2) is composed of two feedback loops: one reinforcing (represented by the letter ‘R’, which is promoting growth) and one balancing loop (represented by the letter ‘B’, which is equilibrating/balancing the system). This kind of model structure composed by one reinforcing loop sided by one balancing loop represents a behavior pattern based on the archetype “limits to growth” or “limits to success” [50].

The resultant baseline model (Figure 2) was then populated with quantitative values derived from the results of both meta-analyses (Tables S4 and S5). We then simulated the model to get an impression of the different behaviors of Brazilian and Indian eligible people for clinical trials.

The model pathway starts with ‘Patients willing to participate in clinical trials’, which is the flow that is regulating the amount of clinical trials of that system. In other words, the more patients willing to participate, the more clinical trials will be accumulated. That flow is regulated by the ratio between ‘Motivations to participate’ (blue arrow, representing positive reinforcement) and ‘Barriers to participate’ (red arrow, representing negative reinforcement).

Following the stock of clinical trials, the system proceeds with the ‘Need to produce more evidence’ which ultimately leads to the ‘Need to recruit more patients to participate in clinical trials’. The higher the amount of conducted clinical trials in a specific country is, the more attractive the country will be for further clinical trials and therefore the “Need to recruit more patients to participate in clinical trials” will increase. Patient recruitment leads again to ‘Motivations to participate’ and ‘Barriers to participate’ completing both reinforcing and balancing loops, respectively. The elements that influence the ‘Motivations to participate’ (convenience, altruism, trust to physicians, monetary reimbursement and personal health benefits) and ‘Barriers to participate’ (fear of side effects, inconvenience, language, mistrust, dependency issues, lack of knowledge and loss of confidentiality) were yielded from the systematic review and meta-analysis results from Brazilian and Indian subjects.

## Supporting Information

Table S1 Characteristics of studies included in meta-analysis.
(0.03 MB DOC)

Table S2 Factors favoring participation in clinical trials.
(0.03 MB DOC)

Table S3 Factors serving as barrier to participation in clinical trials.
(0.03 MB DOC)

Table S4 Summary of factors motivating participation in clinical trials: comparison between Brazilian and Indian people eligible to participate in clinical trials [11].
(0.03 MB DOC)

Table S5 Summary of factors serving as barrier to participation in clinical trials: comparison between Brazilian and Indian people eligible to participate in clinical trials [11].
(0.03 MB DOC)
