# Supplementary material for: So Different, yet So Similar: Meta-Analysis and Policy Modeling of Willingness to Participate in Clinical Trials among Brazilians and Indians
Source: PLoS One. 2010 Dec 16;5(12):e14368. doi: 10.1371/journal.pone.0014368 (PMC3002940; doi:10.1371/journal.pone.0014368)
Supplement: Table S1 — Characteristics of studies included in meta-analysis. (0.03 MB DOC) [file pone.0014368.s001.doc]

**Table S1: Characteristics of studies included in meta-analysis**

| **Study title** | **Country** | **Total Subject Population (Brazilians)** | **Intervention** | **Age group** | **Gender** | **Factors evaluated** | **Factors favoring** | **Factors serving as barrier** |
| --- | --- | --- | --- | --- | --- | --- | --- | --- |
| [Assessment of Knowledge and Attitudes of Young Uninsured Women toward Human Papillomavirus Vaccination and Clinical Trials](http://www.ncbi.nlm.nih.gov/pubmed/16624694) | Salvador , BA, Brazil | 204 | Individual face to face interviews | 16 to 23 years old | All female | Knowledge and attitudes of young uninsured women toward human papillomavirus (HPV) vaccination and clinical trials. | Personal health benefits, altruism, convenience and monetary reimbursement. | Fear of adverse events and inconvenience |
| [Design, Implementation, and Evaluation at Entry of a Prospective Cohort Study of Homosexual and Bisexual HIV-1–Negative Men in Belo Horizonte, Brazil: Project Horizonte](http://www.ncbi.nlm.nih.gov/pubmed/11103049) | Belo Horizonte, MG, Brazil | 470 | Standardized form, given to all volunteers, was composed of five modules | 18 and 59 years | All male | The evaluation of seroincidence of HIV, to ascertain the role of counseling on behavior modification and to assess their willingness to participate in future HIV vaccine trials. | Personal health benefits | Mistrust. |
| [Profile of clinical research subjects in an independent outpatient center](http://www.scielo.br/scielo.php?script=sci_arttext&pid=S1413-81232008000300025&lng=en&nrm=iso) | Rio de Janeiro, RJ, Brazil | 692 | Self-explanatory questionnaire. | NA | All female | The quality of assistance in a clinical trial outpatient center as well as the patients´understanding of the informed consent (IC); determine the reasons why they participate and detail socio-economic levels. | Personal health benefits, altruism and  convenience. |  |
| [Willingness to Participate in HIV Vaccine Trials Among Men Who Have Sex With Men in Rio de Janeiro, Brazil](http://www.ncbi.nlm.nih.gov/pubmed/11141246) | Rio de Janeiro, RJ, Brazil | 927 | Extensive questionnaire | 18 and 50 years | All male | The willingness to participate in HIV vaccine trials of initially HIV-seronegative homosexual men | Personal health benefits and altruism. | Fear of adverse events, mistrust and lack of knowledge. |
| [Willingness to participate in HIV vaccine trials among a sample of men who have sex with men, with and without a history of commercial sex, Rio de Janeiro, Brazil](http://dx.doi.org/10.1080/0954012031000134773) | Rio de Janeiro, RJ, Brazil | 627 | Standardized questionnaire | 18 - 50 years of age | All male | Assess willingness of men who have sex with men (MSM) enrollled in a vaccine preparedness study (‘Projeto Rio’) to participate in phase III anti-HIV/AIDS vaccine trials. | **Personal health benefits and altruism.** | | Fear of adverse events and mistrust. | | --- | |
