# Supplementary material for: So Different, yet So Similar: Meta-Analysis and Policy Modeling of Willingness to Participate in Clinical Trials among Brazilians and Indians
Source: PLoS One. 2010 Dec 16;5(12):e14368. doi: 10.1371/journal.pone.0014368 (PMC3002940; doi:10.1371/journal.pone.0014368)
Supplement: Table S2 — Factors favoring participation in clinical trials. (0.03 MB DOC) [file pone.0014368.s002.doc]

**Table S2: Factors favoring participation in clinical trials**

| **Personal health Benefits** | **Altruism** | **Convenience** | **Monetary reimbursement** |
| --- | --- | --- | --- |
| **·       To have careful/detailed consultations by the same physician**  **·       Access to more information on women’s health**  **·       Potential for their own benefit**  **·       Access to consultation with other medical specialties**  **·       the possibility of getting a free HIV test, because many of them did not have health insurance**  **·       To know more about my health problem**  **·       Because there is no health care in her city**  **·       Receive free bus ticket and snack**  **·       To protect (myself) against HIV-infection**  **·       To enjoy sex without being concerned with AIDS** | **·       Potential for other women benefit**  **·       To benefit other people in the future**  **·       Help the community**  **·       Human concerns/solidarity**  **·       I trust scientific achievements** | **Office visits on time (without having to wait long)**  **·       To have exams and get drugs for free** | **Monetary reimbursement** |
| **30%** | **55%** | **11%** | **6%** |
