# Supplementary material for: So Different, yet So Similar: Meta-Analysis and Policy Modeling of Willingness to Participate in Clinical Trials among Brazilians and Indians
Source: PLoS One. 2010 Dec 16;5(12):e14368. doi: 10.1371/journal.pone.0014368 (PMC3002940; doi:10.1371/journal.pone.0014368)
Supplement: Table S3 — Factors serving as barrier to participation in clinical trials. (0.03 MB DOC) [file pone.0014368.s003.doc]

**Table S3: Factors serving as barrier to participation in clinical trials**

| **Fear of Adverse events** | **Inconvenience** | **Mistrust** | **Lack of knowledge** |
| --- | --- | --- | --- |
| **·       ‘‘fear of adverse events’’**  **·       "Concern about possible adverse effects of the vaccine" "afraid to get AIDS after vaccination"**  **·       Fear of becoming HIV infected from vaccine itself**  **·       Fear of vaccine-induced positive HIV serologic test result**  **·       Fear of vaccine side effects** | **. clinic too far from home’’**  **·       ‘‘gynecologic examination discomfort’’;**  **"Need to get three intramuscular injections"**  **·       "Long duration of the trial (more than 3 years)"**  **·       "More than one visit per year "** | **·       Insecurity**  **·       Afraid to be used as a guinea pig**  **·       Do not want to be a human guinea pig**  **·       Believe that the vaccine will fail**  **·       Do not trust Brazilian government**  **·       Do not trust drug companies**  **·       Do not trust United States**  **·       Do not trust research scientists** | **not having enough information about vaccines’** |
| **12%** | **2%** | **6%** | **4%** |
