# Supplementary material for: So Different, yet So Similar: Meta-Analysis and Policy Modeling of Willingness to Participate in Clinical Trials among Brazilians and Indians
Source: PLoS One. 2010 Dec 16;5(12):e14368. doi: 10.1371/journal.pone.0014368 (PMC3002940; doi:10.1371/journal.pone.0014368)
Supplement: Table S4 — Summary of factors motivating participation in clinical trials: comparison between Brazilian and Indian people eligible to participate in clinical trials [11]. (0.03 MB DOC) [file pone.0014368.s004.doc]

**Table S4 Summary of factors motivating to participate in clincial trials: comparison between Brazilian and Indian people eligible to participate in clinical trials [11]**

|  | Brazilians (%) | Indians (%) |
| --- | --- | --- |
| Personal health benefits | 30 | 48 |
| Altruism | 55 | 43 |
| Convenience | 11 | - |
| Monetary reimbursement | 6 | 31 |
| Trust to Physicians | - | 8 |
