# Supplementary material for: So Different, yet So Similar: Meta-Analysis and Policy Modeling of Willingness to Participate in Clinical Trials among Brazilians and Indians
Source: PLoS One. 2010 Dec 16;5(12):e14368. doi: 10.1371/journal.pone.0014368 (PMC3002940; doi:10.1371/journal.pone.0014368)
Supplement: Table S5 — Summary of factors serving as barrier to participation in clinical trials: comparison between Brazilian and Indian people eligible to participate in clinical trials [11]. (0.03 MB DOC) [file pone.0014368.s005.doc]

**Table S5 -Summary of factors serving as barrier to participate in clinical trials: comparison between Brazilian and Indian people eligible to participate in clinical trials [11]**

|  | Brazilians (%) | Indians (%) |
| --- | --- | --- |
| Fear of side effects | 12 | 27 |
| Inconvenience | 2 | 11 |
| Mistrust | 6 | 26 |
| Lack of knowledge | 4 | - |
| Loss of Confidentiality | - | 17 |
| Dependency Issues | - | 19 |
| Language | - | 1 |
